# Review of Routine Laboratory Monitoring for Patients with Rheumatoid Arthritis Receiving Biologic or Nonbiologic DMARDs

**DOI:** 10.1155/2017/9614241

**Published:** 2017-10-31

**Authors:** William F. C. Rigby, Kathy Lampl, Jason M. Low, Daniel E. Furst

**Affiliations:** ^1^Geisel School of Medicine, Dartmouth College, Lebanon, NH, USA; ^2^Ultragenyx Pharmaceutical, Inc., Novato, CA, USA; ^3^Genentech, Inc., South San Francisco, CA, USA; ^4^Division of Rheumatology, Department of Medicine, David Geffen School of Medicine, University of California, Los Angeles, Los Angeles, CA, USA; ^5^University of Washington, Seattle, WA, USA; ^6^University of Florence, Florence, Italy

## Abstract

Safety concerns associated with many drugs indicated for the treatment of rheumatoid arthritis (RA) can be attenuated by the early identification of toxicity through routine laboratory monitoring; however, a comprehensive review of the recommended monitoring guidelines for the different available RA therapies is currently unavailable. The aim of this review is to summarize the current guidelines for laboratory monitoring in patients with RA and to provide an overview of the laboratory abnormality profiles associated with each drug indicated for RA. Recommendations for the frequency of laboratory monitoring of serum lipids, liver transaminases, serum creatinine, neutrophil counts, and platelet counts in patients with RA were compiled from a literature search for published recommendations and guidelines as well as the prescribing information for each drug. Laboratory abnormality profiles for each drug were compiled from the prescribing information for each drug and a literature search including meta-analyses and primary clinical trials data.

## 1. Introduction

Rheumatoid arthritis (RA) is a chronic systemic inflammatory disease that, without treatment, leads to permanent joint damage and destruction. Patients with RA have an increased risk of comorbidities (most commonly cardiovascular [CV] disease and infection) and decreased survival (the standardized mortality ratio is ≈2, with no decrease observed over time) compared with the general population, and the majority of premature deaths are due to CV disease [1–5]. The higher risk of CV disease in patients with RA is generally thought to be due to the increased inflammatory burden, which causes accelerated atherosclerosis [6], as well as a greater prevalence of traditional risk factors (hypertension, dyslipidemia, and smoking) [1, 7, 8]. The elevated risk of infection in patients with RA may be due either to the immunomodulatory effects of RA itself or to the immunosuppressive effects of RA-related treatments [9]. Patients with RA are also at an increased risk of renal impairment, which is often associated with CV risk factors [10].

Many of the drugs indicated for the treatment of RA can exacerbate comorbidity risks. In addition, the drugs themselves can cause adverse events. Close monitoring of patients receiving treatment for RA is therefore a critical part of patient care. The first line of treatment for RA is with conventional synthetic disease-modifying antirheumatic drugs (csDMARDs), usually methotrexate [11, 12]. However, approximately 50% to 70% of patients have an inadequate response to methotrexate alone [13–15]. In patients who have an inadequate response to csDMARDs, initiation of a biologic DMARD, as monotherapy or in addition to the csDMARD, may provide increased efficacy. Biologics indicated for RA include anti-tumor necrosis factor (aTNF) agents; the B-cell-targeting anti-CD20 monoclonal antibody rituximab; the T-cell costimulatory modulator abatacept; the interleukin 1 receptor antagonist anakinra; and the anti-interleukin 6 alpha receptor monoclonal antibody tocilizumab. Tofacitinib, a targeted small-molecule DMARD that inhibits JAK/STAT signaling, is also available in some countries. Lastly, glucocorticoids are frequently administered to control symptoms of RA.

Safety concerns associated with biologic and nonbiologic DMARDs include increased CV risk, liver and hematologic toxicity, renal impairment, infection, and bleeding. These concerns may be attenuated by identifying abnormal laboratory values early and adjusting medication use accordingly; however, specific recommendations for the frequency of laboratory monitoring in patients with RA are often unclear and left to the treating physician's discretion. There is a need to minimize phlebotomies and physician visits and the inconvenience of patient time spent on them. Because physicians may take into consideration the necessary laboratory monitoring rubrics when choosing between different DMARDs, the purpose of this article is to review the current guidelines for laboratory monitoring in patients with RA, in general, and during treatment. We also set out to provide an overview of the laboratory abnormality profile associated with each drug indicated for RA. Although laboratory testing can refer to much more, such as biomarkers and predictive markers for response, this review is limited to laboratory tests primarily concerned with drug toxicity and pharmacodynamics. Further, this report limits its review to monitoring guidelines for the most common laboratory tests for toxicity: serum lipids, liver aminotransferases, serum creatinine, absolute neutrophil counts (and potential for associated infections), and platelets. Although there is variation in physician views regarding monitoring requirements based on personal experience, this review covers the recommendations given in the prescribing information of each drug as well as by the American College of Rheumatology (ACR), the European League Against Rheumatism (EULAR), and the British Society for Rheumatology (BSR) [11, 12, 16–18].

## 2. Methods

For data on the effects of DMARDs on laboratory measures, PubMed was searched using the name of the agent in question and “rheumatoid arthritis” in combination with terms related to the laboratory measure, such as “lipid”, “cholesterol”, “cardiovascular”, “kidney”, “liver”, “neutrophil”, “neutropenia”, “platelet” and “thrombocytopenia”. Further relevant information was obtained from primary clinical trials, the prescribing information for the DMARDs, and the authors' own experiences.

## 3. Overview of Good Clinical Practice Recommendations for Laboratory Monitoring in Patients With RA 

### 3.1. CV Risk and Serum Lipid Levels

The American Heart Association recommends that people aged 20 years or older and not diagnosed with CV disease have their cholesterol levels checked every 4 to 6 years as part of a cardiovascular risk assessment, and more often if the risk is elevated. Patients with RA have a 50% to 60% increased risk for CV-related death compared with the general population [1, 19]. Because of this increased risk, EULAR guidelines recommend that all patients with RA should undergo an annual CV risk assessment, as treatment and underlying inflammation may alter CV risk factors [7]. However, individual risk profiles will vary; therefore, the EULAR guidelines suggest that the treatment and follow-up plan be determined on an individual basis. It has been noted that CV risk assessment can be easily incorporated into a routine RA visit by adding the determination of nonfasting lipids (total cholesterol [TC], low-density lipoprotein [LDL], and high-density lipoprotein [HDL]) to routine laboratory tests and that the ratio of TC to HDL is the most stable marker of lipid-associated CV risk in RA. Because the TC : HDL ratio does not require fasting, it is also the most convenient method of assessing lipid-associated CV risk [7]. Intervention with statins in patients with RA is recommended at the same frequency as in the general population and in accordance with the national guidelines for the general population [7]. Complicating CV risk assessment and subsequent treatment is the “lipid paradox,” an observed effect in which increased inflammatory burden is associated with decreased serum lipid levels [20, 21]. Because of this paradoxical effect between inflammation and serum lipid levels, a recent review suggested that traditional, lipid profile-based CV disease risk stratification should not be applied to patients with active RA; rather, lipid levels should be assessed after control of inflammation is achieved [20].

### 3.2. Kidney Function Monitoring

Kidney disease is relatively common (~8% to 15%) in patients with RA and may arise as a result of the treatments for RA or the presence of amyloidosis or vasculitis [10, 22]; the contributory role of inflammation in renal impairment remains unclear. Currently, specific guidelines on the recommended frequency of renal function monitoring in patients with RA are scarce, but a study from Couderc et al. on the prevalence of renal dysfunction in RA concluded that, regardless of the treatment regimen, at least annual creatinine measurements appear necessary for patients with RA [10]. This approach is further strengthened by the frequent use of methotrexate and its clearance by the kidney; if abnormal renal function is present, more frequent monitoring is justifiable. Amyloidosis is one of the most severe renal complications of RA, with a reported incidence of 5% to 19% [23]. However, it is likely that this incidence is quite low due to the increase in effective treatment options for RA [24]. Given that the presenting feature of amyloidosis often is proteinuria, testing for proteinuria in patients with longer duration of and/or uncontrolled RA may be prudent [25].

### 3.3. Liver Function

Liver injury is generally not a manifestation of RA. However, medications for RA (usually long-term methotrexate or leflunomide therapy) are associated with abnormal liver function. For this reason, recommendations for monitoring liver enzymes (alanine aminotransferase [ALT] and aspartate aminotransferase [AST]) and performing liver function tests vary depending on individual therapeutic regimens.

### 3.4. Neutropenia

It has been shown that, after adjustment for several key factors, patients with RA are significantly more likely to develop serious infections compared with the general population (hazard ratio, 1.83 [95% CI, 1.52–2.21]) [9]. Severe neutropenia is an uncommon feature of RA (Felty's syndrome), with most cases arising as a consequence of RA-related therapies [26]. Neutropenia has been linked to an increased risk of infection; however, it is important to note that cases of therapy-associated severe neutropenia have been observed without incidence of infection [27]. Because the risk of infection may increase with the severity and duration of neutropenia, routine monitoring of absolute neutrophil count (ANC) is necessary during RA treatment; recommendations for the frequency of monitoring ANCs are specific to individual medications.

### 3.5. Thrombocytopenia

Although thrombocytopenia is an uncommon feature of RA, its occurrence may be associated with RA-related therapies. Although cases of drug-induced thrombocytopenia have been associated with clinically important bleeding events [28], the association of bleeding events with RA therapy-induced thrombocytopenia is not known. Monitoring of platelet counts is important during RA treatment to assess the risk of internal bleeding, and, as with ANCs, the recommendations for the frequency of monitoring platelets in patients with RA are specific to the individual therapeutic regimen.

## 4. Monitoring Guidelines for Conventional Synthetic, Biologic, and Targeted Small-Molecule DMARDs in Patients with RA

Laboratory monitoring guidelines for serum lipids, liver enzymes, serum creatinine, neutrophils, and platelets in patients receiving DMARDs are described in the prescribing information for each drug, drug reports from the manufacturers, experts in the field, the ACR recommendations for laboratory monitoring in patients with RA during treatment with DMARDs, and the BSR guideline for DMARD therapy (Table 1) [16–18]. The EULAR guidelines for managing RA with biologic and conventional DMARDs do not make specific recommendations regarding the frequency of routine laboratory monitoring [12].

### 4.1. Conventional Synthetic DMARDs

There are no specific guidelines in place for monitoring lipid levels during csDMARD therapy (Table 1). With regard to liver toxicity, the ACR recommends that, for patients receiving methotrexate, leflunomide, or sulfasalazine, liver enzymes should be measured at baseline, every 2 to 4 weeks for the first 3 months, every 8 to 12 weeks for the 3 to 6 months after initiation, and every 12 weeks thereafter [16]. This is slightly less stringent than the guidelines given in the prescribing information for methotrexate and leflunomide, which recommend liver enzyme and function tests every 1 to 2 months throughout methotrexate therapy and monitoring of ALT levels every 6 to 8 weeks throughout leflunomide therapy [29, 30]. The BSR recommends that the frequency of ALT and/or AST monitoring during methotrexate and leflunomide therapy falls between these ranges, with the specification that after 3 months of a stable dose of methotrexate or leflunomide, the monitoring be reduced to every 12 weeks, with more frequent monitoring in patients at higher risk of toxicity [17, 18].

Because methotrexate, leflunomide, and sulfasalazine all suppress cell proliferation, routine hematologic monitoring is necessary [18]. For these drugs, the ACR recommends complete blood counts at baseline, every 2 to 4 weeks for the first 3 months, every 8 to 12 weeks for months 3 to 6, and every 12 weeks thereafter [16]. These guidelines are also less stringent than those given in the prescribing information for methotrexate and leflunomide, which recommend a complete blood count with differential and platelet counts at least monthly throughout methotrexate therapy and platelet and white blood cell counts every 6 to 8 weeks throughout leflunomide therapy [29, 30]. The BSR recommendations again fall between these ranges, with the specification that a full blood count be performed every 2 weeks during methotrexate therapy until the dose is stable for 6 weeks, then monthly for 3 months, and at least every 12 weeks thereafter [17, 18].

If combinations of csDMARDs are considered, most guidelines suggest following the most stringent laboratory testing among the drugs being combined.

### 4.2. Biologic and Targeted Synthetic DMARDs: Liver and Lipid Monitoring

Among the biologic and targeted synthetic DMARDs, tocilizumab and tofacitinib are the only ones for which specific recommendations for monitoring serum lipids and liver function are given (Table 1). According to the prescribing information, lipids (TC, triglycerides, LDL-C, and/or HDL-C) should be measured 4 to 8 weeks after initiation for both tocilizumab and tofacitinib and every 24 weeks thereafter for tocilizumab [31, 32]. For tocilizumab, ALT and AST levels should be measured 4 to 8 weeks after initiation and every 3 months thereafter. The prescribing information for tofacitinib states that there should be routine monitoring of all liver enzymes [32].

### 4.3. Biologic and Targeted Synthetic DMARDs: Neutrophil and Platelet Count Monitoring

Biologic DMARDs are often associated with transient, sustained, or late-onset decreases in neutrophils and/or platelets. The prescribing information for both tocilizumab and tofacitinib suggests monitoring of ANCs 4 to 8 weeks after initiation and every 3 months thereafter [31, 32]. For rituximab, continued monitoring of complete blood counts, including ANCs, is recommended at 2- and 4-month intervals during rituximab therapy [33].

### 4.4. Biologic and csDMARD Combination Therapy

A recent, large observational study showed that, among patients with RA initiating biologic therapy, >70% of patients initiated the biologic in combination with a csDMARD [34, 35]. Concomitant methotrexate is often part of the therapeutic regimen for patients receiving aTNF agents, as it can reduce the development of anti-drug antibodies and increase the efficacy of aTNF therapy in the treatment of RA [36–38]. In particular, it has been shown that concomitant administration of methotrexate suppresses development of anti-adalimumab and anti-infliximab antibodies, maximizing efficacy and reducing the occurrence of certain adverse drug reactions [39, 40]. Laboratory monitoring for patients receiving combination therapy should follow the guidelines for both the biologic and csDMARD(s).

### 4.5. Glucocorticoids

Despite conflicting information on the relationship between glucocorticoids and dyslipidemia, Liu et al. recommend regular monitoring of lipids in patients receiving glucocorticoids (including low doses) for prolonged periods or at high doses: for patients scheduled for long-term systemic corticosteroid therapy, serum lipid levels should be assessed at baseline, 1 month after glucocorticoid initiation, and then every 6 to 12 months thereafter [41]. No recommendations are in place for monitoring of liver function, neutrophils, or platelets for patients receiving glucocorticoids.

## 5. Profiles of Laboratory Abnormalities and Associated Clinical Sequelae in Patients with RA

### 5.1. Lipids

Despite an increased risk of CV events in patients with RA, growing evidence suggests that patients with active, untreated RA have* lower* serum TC and LDL-C levels than the general population [20]. Serum lipid levels in patients with RA can respond to changes in the inflammatory burden as well as to DMARD therapy and/or glucocorticoid therapy (Table 2). Consistent with this paradox in lipid levels and disease activity, achievement of reduced inflammation with DMARD therapy often results in increased serum lipid levels [20, 21].

Most csDMARDs, including methotrexate and sulfasalazine, are associated with increased TC, LDL-C, and HDL-C levels (Table 2) [42, 43]. Hydroxychloroquine/chloroquine, however, has been reported to decrease TC and LDL-C levels, which is consistent with previously reported associations between antimalarials and favorable lipid profiles [44].

The degree to which biologic therapy affects serum lipid levels is complex, with differing reports; the effect of a biologic or targeted nonbiologic therapy itself on lipid levels is also complicated by the frequent administration of concurrent csDMARDs, which limits the ability to compare the effects between therapies (Table 2) [34, 35]. Tocilizumab and tofacitinib have been shown to increase TC and LDL-C and, to a lesser extent, HDL-C in patients with RA [27, 45, 46]. In a long-term (up to 4.6 years) study of pooled tocilizumab clinical trials, TC and LDL-C levels increased by week 6 and remained relatively stable at subsequent time points [27]. The MEASURE study demonstrated that tocilizumab induced quantitative changes in lipoprotein profiles, including elevations in LDL-C, but also altered HDL particles towards an anti-inflammatory composition, whereby proinflammatory HDL-associated serum amyloid A and secretory phospholipase A2 significantly decreased from baseline during 24 weeks of tocilizumab treatment [47]. An analysis of pooled tofacitinib clinical trials in patients with RA demonstrated dose-dependent increases in serum TC, LDL-C, and HDL-C within the first 1 to 3 months of therapy, which remained stable thereafter [45]. A recent meta-analysis demonstrated that patients with RA treated with aTNF agents had a significant increase in HDL-C in the first 2 to 6 weeks of therapy, after which HDL-C remained stable [48]. Despite modest increases in TC and LDL-C levels with aTNF use at 6 months, there was no significant overall effect on the atherogenic index [48]. Overall, the trend towards elevated TC and LDL-C levels in patients with RA appears consistent among the different biologic and targeted nonbiologic therapies; however, there is no apparent associated increase in the risk of atherosclerosis, and some studies have suggested that biologic therapy actually reduces CV risk [45, 49–52].

The relationship between lipid levels and CV risk in patients with RA receiving DMARDs is not well understood. In studies that measured lipid levels following conventional synthetic, biologic, or targeted small-molecule DMARD therapy, there was no evidence that the elevated lipid levels with any of the DMARDs were associated with increased risk of CV disease, and indeed the reverse may be true: a large, prospective study of 1240 patients with RA demonstrated that methotrexate use significantly reduced the risk of CV mortality [53]. Additionally, results from the Corrona registry demonstrated that patients with RA who received aTNF agents had a reduced risk of CV events compared with patients who received csDMARDs; however, glucocorticoid use, which may be a surrogate marker of increased inflammation, was associated with a dose-dependent increase in risk of CV events [50].

Glucocorticoids have varying effects on lipid levels in patients with RA. In a prospective study of 42 patients with newly diagnosed RA being treated with csDMARDs, there was no significant difference in lipid levels at 12 months between corticosteroid users and nonusers [54]. In contrast, in patients with RA from the COBRA trial treated with sulfasalazine monotherapy or sulfasalazine plus methotrexate with or without high but rapidly tapered prednisone, HDL-C levels increased by 50%; this elevation occurred much more quickly in steroid users than nonusers (16 and 40 weeks, resp.) [55].

### 5.2. Liver Enzymes

Increases in liver aminotransferases have been noted in patients with RA following administration of DMARDs (Table 3). Among the csDMARDs, methotrexate and leflunomide are most frequently associated with elevations in liver enzymes; this association correlates with longer duration of use. In a controlled trial comparing safety and efficacy of methotrexate versus that of leflunomide in patients with RA, 999 patients were randomized to leflunomide (*n* = 501) or methotrexate (*n* = 498) and followed for 2 years [56]. Of these patients, 25% of patients who received methotrexate and 6.4% of patients who received leflunomide had elevated ALT and AST levels > 3 × upper limit of normal (ULN) during the first year (alcohol use was not accounted for in this study). Over the 2 years, 4.2% of patients discontinued methotrexate and 1.6% discontinued leflunomide due to elevated serum liver enzymes. This is comparable to the results of a systematic literature review that demonstrated that 3.7% of patients with RA who received methotrexate discontinued due to liver toxicity (mean treatment duration of 55.8 months and mean dose of 10.5 mg/week) [57].

Most published reports of elevated liver enzymes in patients receiving biologic or targeted synthetic DMARDs have involved patients who received concomitant methotrexate or leflunomide. In studies of patients who received biologic DMARDs, higher percentages of patients had elevations in AST or ALT with coadministration of methotrexate than with administration of biologic monotherapy (Table 3). For example, among patients who received golimumab + methotrexate combination therapy in a phase 3 trial, 4% to 6% had elevations in ALT and AST, respectively, whereas, among patients who received golimumab monotherapy, none had any elevation in AST or ALT [58]. In the tocilizumab ACT-RAY trial, 7.7% of patients who received tocilizumab + methotrexate experienced ALT > 3 × ULN by 6 months, whereas 1.2% of patients who received tocilizumab monotherapy experienced ALT > 3 × ULN [59]. In the tofacitinib monotherapy trials, there was no difference in the proportion of patients experiencing increases in liver enzymes > 3 × ULN among those who received tofacitinib monotherapy compared with those who received placebo [32].

### 5.3. Neutrophils

Neutropenia is a common adverse event in patients receiving treatment for RA, with the main complication being infection due to bacteria and/or fungi [26]. Mild to moderate decreases in ANCs are often associated with DMARD therapy. With the exception of rituximab, acquired neutropenia in patients with RA receiving biologic or csDMARD therapy is generally transient, with ANCs recovering within a few days of treatment and rarely leading to infection.

Reports of grade 3 or 4 neutropenia (based on the Common Terminology Criteria for Adverse Events) in patients who received csDMARDs are scarce. For clinical trials in which neutropenia was reported, the proportions of patients experiencing neutropenia are shown in Table 4. In a recent RA clinical trial with a methotrexate monotherapy arm, 9.8% of patients who received methotrexate monotherapy experienced grade 1 or 2 neutropenia (grades 1 and 2 are described as mild and moderate, resp.), but only 0.4% experienced grade 3 neutropenia (described as severe or medically significant but not immediately life-threatening) and 0% experienced grade 4 neutropenia (described as life-threatening or disabling) [60]. In a study of patients with juvenile RA who received methotrexate or leflunomide, <1% experienced grade 3 or 4 decreases in neutrophil counts by week 16 [61].

Among studies reporting ANCs following administration of biologic DMARDs, <1% of patients who received adalimumab or etanercept in combination with methotrexate were reported to experience grade 3 or 4 decreases (Table 4) [62, 63]. In the pooled long-term (up to 4.6 years) safety analysis of the tocilizumab RA trials, 4.8% of patients experienced grade 3 decreases in ANCs, and <1% of patients (14 out of 4009 observed patients) experienced grade 4 decreases in ANCs; 1 patient experienced a serious infection of empyema temporally associated with grade 3 neutropenia, and no patients with grade 4 neutropenia experienced serious infection within 30 days of observed neutropenia [27]. In a Phase III clinical trial, 1.1% to 1.6% of patients who received tofacitinib experienced grade 2 or 3 neutropenia at month 3 [62]. Reports of neutropenia in patients with autoimmune disease treated with rituximab are infrequent; however, there have been reports of late-onset neutropenia in 1.3% to 5.8% of patients with RA who received rituximab, observed at medians of 21 to 23 weeks, with variable incidences of associated infections [64–66]. Thus, the relationship between ANC and infection risk may differ among various biologics.

### 5.4. Platelets

In addition to causing decreases in ANCs, DMARD treatment can lead to a transient decrease in platelet counts that generally recovers after 1 week [67]. In clinical trials, <1% of patients have been reported to experience grade 3 or higher (severe/medically significant or worse) decreases in platelet counts with conventional synthetic, biologic, or targeted small-molecule DMARDs (Table 5).

### 5.5. Serum Creatinine

For monitoring and assessment of kidney function, serum creatinine may be measured as opposed to creatinine clearance. The prescribing information for cyclosporine and tacrolimus both notes elevated serum creatinine following administration and recommends close monitoring of renal function. Among the DMARDs discussed here, only tofacitinib has prescribing information that reports drug-associated increases in serum creatinine levels. The mean increase in serum creatinine in patients treated with tofacitinib in clinical trials was <0.1 mg/dL over 12 months of treatment. In the long-term extensions, however, up to 2% of patients discontinued tofacitinib due to an increase in creatinine > 50% above baseline. The clinical significance of the observed serum creatinine elevations is unknown [32].

## 6. Discussion

This review is among the first to comprehensively examine the differences in laboratory monitoring recommendations for each DMARD indicated for the treatment of RA. In particular, guidelines for monitoring serum lipids, liver aminotransferases, serum creatinine, and neutrophil and platelet counts were summarized. Information was gathered from the prescribing information for each drug and from ACR, BSR, and EULAR recommendations. Furthermore, an overview of available information on the laboratory abnormality profiles associated with each drug indicated for RA was given.

Regardless of the choice of treatment, good clinical practice dictates that routine laboratory monitoring for patients with RA is important. Due to the increased risk of CV disease, it is recommended that patients with RA have at least yearly monitoring of serum lipids [68]. In addition, due to the high prevalence of renal impairment in patients with RA, once-yearly monitoring of kidney function is also advised [10].

RA therapies have the potential to exacerbate CV and renal comorbidities; in addition, many drugs indicated for RA have inherent risks associated with them. Although it is recommended that patients with RA be monitored for CV risk (including serum lipid levels) at least yearly, serum lipid levels should be assessed more frequently in those receiving tocilizumab or tofacitinib [31, 32]. When determining an appropriate monitoring regimen, consideration should be given to the observation that high-grade inflammation in patients with active RA is associated with reductions in the levels of TC, HDL-C, and LDL-C; this is seen in other inflammatory states (e.g., autoimmune and sepsis) and is at least partially reversible with anti-inflammatory treatment [69]. Whereas levels of serum lipids tend to predict CV disease risk in the general population, an opposite relationship has been observed in patients with RA: a 2011 retrospective cohort study showed that, in patients with RA, lower levels of TC and LDL-C were associated with* increased* risk of CV disease [70]. Inflammation itself is associated with lower lipid levels, and therefore baseline levels (prior to treatment) may be relatively low, and although lipid levels may increase with DMARD treatment, they often stay within the acceptable reference range. Because of this relationship, the increases in lipids associated with particular biologic DMARDs may actually be a reflection of their efficacy in reducing inflammation, and the use of statins to reduce the associated increase in cholesterol is recommended.

Liver toxicity can be a significant problem with long-term use of methotrexate or leflunomide, warranting frequent monitoring of liver function, particularly ALT and AST levels. Although it may appear that, due to elevations in lipid levels, the recommendations for patients receiving tocilizumab suggest more frequent laboratory monitoring than those for patients receiving aTNF therapy, this is not necessarily true. The aTNF agents are usually administered in combination with methotrexate to reduce immunogenicity and increase efficacy, and infliximab and golimumab are indicated for patients with RA only when administered in combination with methotrexate. In these patients, the monitoring frequency that is recommended for liver toxicity or decreases in ANC due to methotrexate is advised (Table 6).

Although high-dose glucocorticoid treatment for short-term duration is generally considered to be safe, glucocorticoid-associated toxicity is related to both the average daily dose and the lifetime cumulative dose. In a chronic disease such as RA, the frequent use of even low-dose glucocorticoids over long periods commonly reaches cumulative dose thresholds that predispose patients to increased risk of adverse events and mortality [41, 71, 72]. The risks associated with approaching the cumulative glucocorticoid dose thresholds in patients who experience an increase in disease activity (“flare”) while receiving a biologic DMARD should be weighed when considering addition of chronic low-dose glucocorticoid treatment versus a short-term course of high-dose glucocorticoids (dose and duration). It may be prudent for clinicians to keep a record of their patients' cumulative glucocorticoid dose over time.

While this review provides a comprehensive overview of the laboratory monitoring recommendations for drugs indicated for RA, there are some important limitations. All potential guidelines were not reviewed (i.e., liver and renal subspecialty guidelines), which may add to the diversity of recommendations. Neither a systematic literature review nor a meta-analysis was done; this is a descriptive article, as we were interested in those recommendations most familiar to rheumatologists. Further, recommendations were made based on our personal opinions and our reviews to help readers, but each reader is encouraged to reach their own considered opinion. The overview and comparison of guidelines presented here may help to inform and assist physicians in the choices of treatment based on the necessity of laboratory monitoring. Ultimately, what is best for the patient regarding the recommendations and individual patient-risk factors must be considered in all cases.

In closing, the frequency of drug-related toxicities associated with RA therapy is low and therefore easily managed in the vast majority of patients. Although screening guidelines exist, clear evidence of the benefit of monitoring in preventing harm to the patient is lacking. Moreover, does the prior experience of the patient shape the need for laboratory monitoring? For instance, if a patient is stably controlled on methotrexate for 2 years with testing every 2 to 3 months, do they still require that frequency of testing? If not, what frequency is required? In the United States, for a patient with limited means and a high deductible, lab work alone is likely to constitute their major out-of-pocket expense. As a result, it is often difficult to get these patients to agree to be tested more than once or twice a year. Similarly, if someone is on etanercept monotherapy without signs of infection, what frequency of lab testing, if any, is required? While guidelines exist, it is not clear that their rigor has been amply demonstrated. These questions are ones that we await answers to in the coming years.

## Figures and Tables

**Table 1:** Laboratory monitoring guidelines for DMARDs in patients with RA.

	Lipids	Liver enzymes (function, ALT, and AST)	Neutrophils and/or platelets	Serum creatinine	Ref(s)
*Conventional synthetic DMARDs*					

Methotrexate	N/A	LFT every 1-2 months	CBC with differential and platelet counts at least monthly	N/A	PI [29]
	N/A	ALT and AST at baseline, every 2–4 weeks for the first 3 months, every 8–12 weeks the following 3–6 months, and every 12 weeks thereafter	CBC at baseline, every 2–4 weeks for the first 3 months, every 8–12 weeks for 3–6 months after initiation, and every 12 weeks thereafter	At baseline, every 2–4 weeks for the first 3 months, every 8–12 weeks the following 3–6 months, and every 12 weeks thereafter	ACR [16]
	N/A	ALT and/or AST every 2 weeks until stable dose for 6 weeks, then monthly for 3 months; at least every 12 weeks thereafter	Full blood count every 2 weeks until stable dose for 6 weeks, then monthly for 3 months; at least every 12 weeks thereafter	Creatinine/calculated GFR every 2 weeks until stable dose for 6 weeks, then monthly for 3 months; at least every 12 weeks thereafter	BSR [17, 18]

Leflunomide	N/A	ALT levels ≥ monthly for 6 months after initiation; every 6–8 weeks thereafter	Platelet count, white blood cell count, and hemoglobin or hematocrit monitored at baseline and monthly for 6 months after initiation and every 6–8 weeks thereafter	N/A	PI [30]
	N/A	ALT and AST at baseline, every 2–4 weeks for the first 3 months, every 8–12 weeks for 3–6 months after initiation, and every 12 weeks thereafter	CBC at baseline, every 2–4 weeks for the first 3 months, every 8–12 weeks for 3–6 months after initiation, and every 12 weeks thereafter	At baseline, every 2–4 weeks for the first 3 months, every 8–12 weeks for 3–6 months after initiation, and every 12 weeks thereafter	ACR [16]
	N/A	ALT and/or AST every 2 weeks until stable dose for 6 weeks, then monthly for 3 months; at least every 12 weeks thereafter	Full blood count every 2 weeks until stable dose for 6 weeks, then monthly for 3 months; at least every 12 weeks thereafter	Creatinine/calculated GFR every 2 weeks until stable dose for 6 weeks, then monthly for 3 months; at least every 12 weeks thereafter	BSR [17, 18]

HCQ/CQ	N/A	ALT and AST at baseline and none thereafter	CBC at baseline and none thereafter	At baseline	ACR [16, 73]

Gold	N/A	ALT and/or AST every 2 weeks until stable dose for 6 weeks, then monthly for 3 months; at least every 12 weeks thereafter	Full blood count every 2 weeks until stable dose for 6 weeks, then monthly for 3 months; at least every 12 weeks thereafter	Creatinine/calculated GFR every 2 weeks until stable dose for 6 weeks, then monthly for 3 months; at least every 12 weeks thereafter	BSR [17, 18]

Sulfasalazine	N/A	LFT at baseline and every 2 weeks during the first 3 months, monthly during the second 3 months, and every 3 months or as needed thereafter	CBC with differential at baseline and every 2 weeks during the first 3 months, monthly during the second 3 months, and every 3 months or as needed thereafter	N/A	PI [74]
	N/A	ALT and AST at baseline, every 2–4 weeks for the first 3 months, every 8–12 weeks for 3–6 months after initiation, and every 12 weeks thereafter	CBC at baseline, every 2–4 weeks for the first 3 months, every 8–12 weeks for 3–6 months after initiation, and every 12 weeks thereafter	At baseline, every 2–4 weeks for the first 3 months, every 8–12 weeks for 3–6 months after initiation, and every 12 weeks thereafter	ACR [16]
	N/A	ALT and/or AST every 2 weeks until stable dose for 6 weeks, then monthly for 3 months; at least every 12 weeks thereafter	Full blood count every 2 weeks until stable dose for 6 weeks, then monthly for 3 months; at least every 12 weeks thereafter	Creatinine/calculated GFR every 2 weeks until stable dose for 6 weeks, then monthly for 3 months; at least every 12 weeks thereafter	BSR [17, 18]

*Biologic DMARDs* ^d^					

Adalimumab^a^	N/A	N/A	N/A	N/A	

Infliximab^a,b^	N/A	Same as for MTX^b^	Same as for MTX^b^	Same as for MTX^b^	

Etanercept^a^	N/A	N/A	N/A	N/A	

Golimumab^a,b^	N/A	Same as for MTX^b^	Same as for MTX^b^	Same as for MTX^b^	

Certolizumab^a^	N/A	N/A	N/A	N/A	

Tocilizumab	4–8 weeks after initiation and every 24 weeks thereafter	ALT and AST levels 4–8 weeks after initiation and every 3 months thereafter	ANC 4–8 weeks after initiation and every 3 months thereafter	N/A	PI [75]

Rituximab^b^	N/A	Same as for MTX^b^	CBC and platelet counts at 2- and 4-month intervals during rituximab therapy	N/A	PI [33]

Abatacept^a^	None required	None required	N/A	N/A	PI [76]

Anakinra^a^	N/A	N/A	N/A	N/A	

*Targeted small-molecule DMARDs*					

Tofacitinib	4–8 weeks after initiation	Routine monitoring of all	At initiation, 4–8 weeks after initiation, and every 3 months thereafter	N/A	PI [32]

Glucocorticoids	At baseline, 1 month after initiation, and every 6–12 months thereafter	N/A^c^	N/A^c^	N/A^c^	Liu et al. [41]

ACR, American College of Rheumatology; ALT, alanine aminotransferase; AST, aspartate aminotransferase; BSR, British Society for Rheumatology; CBC, complete blood count; DMARD, disease-modifying antirheumatic drug; HCQ/CQ, hydroxychloroquine/chloroquine; LFT, liver function test; MTX, methotrexate; N/A, not available; PI, prescribing information; RA, rheumatoid arthritis; ref, reference. ^a^Adalimumab, infliximab, etanercept, golimumab, certolizumab, abatacept, and anakinra do not currently have a laboratory monitoring program; patients receiving these medications should follow the laboratory monitoring guidelines for any coadministered medications. ^b^Infliximab, golimumab, and rituximab are indicated for RA only in combination with methotrexate. ^c^Glucocorticoid-associated toxicity is dependent on lifetime cumulative dose and average daily dose. ^d^At the time of this review, monitoring guidelines for baricitinib and sarilumab have not been established.

**Table 2:** Effects of nonbiologic and biologic DMARDs on lipid levels in patients with RA.

	Cholesterol			TGs	Ref(s)
	TC	LDL	HDL		
*Conventional synthetic DMARDs*					
Methotrexate	↑ or =	↑ or =	↑	N/A	[42]
Hydroxychloroquine/chloroquine	↓ or =	↓ or =	↑ or =	↓	[44]
Sulfasalazine	↑	=	↑	N/A	[43]

*Biologic DMARDs* ^a^					
Adalimumab	↑ or =	=	↑	=	[77]
Infliximab	↑	↑	↑	↑ or =	[68]
Etanercept	↓	↑ or ↓	↑ or ↓	↓	[77, 78]
Golimumab	↑	↑	↑	↑	[79]
Tocilizumab	↑	↑	=	↑	[31, 77]
Rituximab^b^	N/A	N/A	N/A	N/A	
Abatacept	↑ or =	=	↑	↑	[80]

*Targeted small-molecule DMARD*					
Tofacitinib	↑	↑	↑	N/A	[32, 81]

=, no change; ↑, increase; ↓, decrease; DMARD, disease-modifying antirheumatic drug; HDL, high-density lipoprotein; LDL, low-density lipoprotein; N/A, not available; RA, rheumatoid arthritis; ref, reference; TC, total cholesterol; TG, triglyceride. ^a^No data were available for leflunomide, anakinra, or certolizumab. ^b^Lipid and cholesterol levels were not studied in the RA rituximab clinical trials.

**Table 3:** Proportions of patients who experienced increases in liver enzymes during RA treatment^a^.

% of patients^b^	≤6 months	>6 months	Clinical sequelae
	ALT or AST > 3 × ULN	ALT or AST > 3 × ULN	
*Conventional synthetic DMARDs*			

Methotrexate	**3.6%** [60]	>2 × ULN: **12.9%** [57] **25%** [56]	**3.7%** of patients discontinued due to liver toxicity [57] **4.2%** discontinued by 2 years due to liver toxicity [56]

Leflunomide	>5 × ULN: **3.0%** [82]	**1.5%**–**6.4%** [30, 56]	Due to grade 2 hepatotoxicity, 1 patient (1%) was withdrawn from LEF treatment and 1 patient (1%) continued LEF at a reduced dose [82]. 1.6% of patients discontinued by 2 years [56]

LEF + MTX	**6.8%** [83]	**3.8%** for LEF + MTX; (**0.8%** for MTX + placebo) [30] **17%** [83]	Four patients (4.5%) were withdrawn from treatment due to persistent elevation of plasma liver enzyme level

Sulfasalazine	**4%** [84]	N/A	N/A

*Biologic DMARDs* ^c^			

Adalimumab	**3.5%** ^d^ [85] **<1%** (w/MTX) [62]	**<1%** (w/MTX) [62]	N/A

Infliximab	>5 × ULN: **<1%**^e^	≥3 × ULN: **5%** (w/MTX)^c^	N/A

Etanercept	Grades 2–4: **0%** [86]	Grades 2–4: **0%** [86]	N/A

Golimumab	Any ALT or AST elevation: **0**% (monotherapy); **4.4%**–**6.3%** (w/MTX) [58]	N/A	N/A

Tocilizumab	**3.4%**–**6.5%** [31, 87]	**11%** (TCZ + MTX); **2%**-**3%** (MTX → TCZ) [88] **10.8%** [27]	There were no serious liver function disorders during TCZ treatment [89]. Seven patients (1.1%) prematurely discontinued due to elevated liver aminotransferases (with concomitant MTX) [87]

Anakinra	N/A	Patients with elevated liver enzymes: **<1%** [90]	N/A

*Targeted small-molecule DMARD*			

Tofacitinib	**1.1%**–**1.6%** (w/MTX) [91] **≤1.0%** [92]	**<1%** [91] **3.2%** (w/MTX) [62]	N/A

ALT, alanine aminotransferase; AST, aspartate aminotransferase; DMARD, disease-modifying antirheumatic drug; LEF, leflunomide; MTX, methotrexate; N/A, not available; RA, rheumatoid arthritis; TCZ, tocilizumab; ULN, upper limit of normal. ^a^For ALT and AST levels, grade 3 was >5 to 10 × ULN and grade 4 was >10 × ULN. ^b^Data are the proportions of patients with ALT or AST ≥ 3 × ULN except where noted. ^c^No data were available for rituximab, certolizumab, or abatacept. ^d^Many of these patients were also taking methotrexate or nonsteroidal anti-inflammatory drugs. ^e^Company medical letter (personal communication), data on file.

**Table 4:** Proportions of patients who experienced neutropenia during RA treatment in clinical trials^a^.

% of patients^b^	≤6 months	>6 months	Clinical sequelae
	Grade 3 or 4	Grade 3 or 4	
*Conventional synthetic DMARDs* ^c^			

Methotrexate	**0.4%** [60]	N/A	N/A

Leflunomide	Grade 4: **0%** [61]	N/A	N/A

Sulfasalazine	N/A	**0%** [63]	N/A

*Biologic DMARDs* ^c^			

Adalimumab	Grade 2 or 3: **<1.0%** [62]	**≤1.0%** [62]	N/A

Infliximab	N/A	Grade 2, 3, or 4: **1.7%**–**8.8%**^d^ [93]	N/A

Etanercept	N/A	**0%** [63]	N/A

Golimumab	≥1 abnormal value: **2.3%**^e^	N/A	N/A

Tocilizumab	Grade 3:** 4.8%** [27] Grade 4:** <1.0%** [27]	N/A	One patient experienced a serious infection of empyema temporally associated with grade 3 neutropenia. None of the patients with grade 4 neutropenia experienced serious infection within 30 days of observed neutropenia [27]

Rituximab	LON: **4.6%**–**6%** [64, 65]	N/A	LON was defined as an ANC < 1.0 × 10^9^/L occurring 4 weeks after the last rituximab infusion. Most cases resolved spontaneously or following administration of G-CSF or GM-CSF. Febrile neutropenia and neutropenia-related infections requiring hospitalization have been observed

Abatacept	N/A	N/A	N/A

Anakinra	**0.4%** [94]	**0.5%** [90]	While neutropenic, 1 patient developed cellulitis. In most cases, grade 3 or 4 toxic events were sporadic and were not associated with progressive decreases nor were indicative of drug-related impairment [90]

*Targeted small-molecule DMARDs*			

Tofacitinib	**<1%** [92] Grade 2 or 3: **0.9%**–**3.1%** [60, 91, 92] Grade 4: **0%** [91]	Grade 2 or 3: **1.3%** [92]	N/A

ANC, absolute neutrophil count; DMARD, disease-modifying antirheumatic drug; G-CSF, granulocyte colony-stimulating factor; GM-CSF, granulocyte-macrophage colony-stimulating factor; LON, late-onset neutropenia; N/A, not available; RA, rheumatoid arthritis; TCZ, tocilizumab. ^a^Neutropenia grades were defined as follows: grade 2, ≥1000 to <1500 cells/mm^3^; grade 3, ≥500 to <1000 cells/mm^3^; grade 4, <500 cells/mm^3^. ^b^Data are the proportions of patients experiencing grade 3 or 4 ANCs except where noted. ^c^Data were not available for hydroxychloroquine/chloroquine or certolizumab. ^d^From a cohort of patients with polyarticular juvenile idiopathic arthritis who received infliximab plus methotrexate. ^e^Company medical letter (personal communication), data on file.

**Table 5:** Proportions of patients who experienced decreases in platelet counts during RA treatment in clinical trials^a^.

% of patients^b^	≤6 months	>6 months	Clinical sequelae
	Grade 3 or 4	Grade 3 or 4	
*Conventional synthetic DMARDs* ^c^			

Methotrexate	**0%**–**1.3% **[61, 67, 95]	N/A	N/A

Leflunomide	**0% **[61]	**0%** ^d^ [96]	N/A

*Biologic DMARDs* ^c^			

Adalimumab	**0.2%** ^e^	N/A	N/A

Infliximab	N/A	**0%** ^d^ [96]	N/A

Golimumab	≥1 abnormal value: **0.2%**^b^	N/A	N/A

Tocilizumab	**0.2%** [87]	**<1.0% **[27]	One serious bleeding event of hemorrhagic stomatitis occurred in a patient with grade 4 thrombocytopenia [27]

Rituximab	N/A	N/A	N/A

Abatacept	N/A	N/A	N/A

Anakinra	**0%** [94]	**0% **[94]	N/A

DMARD, disease-modifying antirheumatic drug; N/A, not available; RA, rheumatoid arthritis; ^a^Thrombocytopenia grades were defined as follows: grade 2, 50,000 to <75,000 cells/mm^3^; grade 3, 25,000 to <50,000 cells/mm^3^; grade 4, <25,000 cells/mm^3^. ^b^Data are the proportions of patients experiencing grade 3 or 4 platelet counts except where noted. ^c^No data were available for sulfasalazine, hydroxychloroquine/chloroquine, certolizumab, etanercept, or tofacitinib. ^d^Patients received leflunomide plus infliximab. ^e^Company medical letter (personal communication), data on file.

**Table 6:** Summary of recommended frequencies of laboratory monitoring for patients with RA receiving DMARDs^a^.

	Lipids	AST and ALT	Neutrophils and platelets
MTX, LEF, SSZ	—	Initially: every 2–4 weeks After ~1–3 months: every 1–3 months After ~6–2 months: every 3 months or based on clinical judgment	Initially: every 2–4 weeks After ~1–3 months: every 1–3 months After ~6–12 months: every 3 months or based on clinical judgment

GLU	Initially: 1 month after initiation After 1 month: every 6–12 months	—	—

TCZ	Initially: 4–8 weeks after initiation After 1–2 months: every 6 months	Initially: 4–8 weeks after initiation After 1-2 months: every 3 months	Initially: 4–8 weeks after initiation After 1-2 months: every 3 months

TOF	Initially: 4–8 weeks after initiation	Routine	Initially: 4–8 weeks after initiation After 1-2 months: every 3 months

RTX	—	—	Every 2-3 months

aTNF	—	aTNFs administered in combination with MTX should follow the MTX monitoring guidelines	aTNFs administered in combination with MTX should follow the MTX monitoring guidelines

ALT, alanine aminotransferase; AST, aspartate aminotransferase; aTNF, anti-tumor necrosis factor agent; DMARD, disease-modifying antirheumatic drug; GLU, glucocorticoid; LEF, leflunomide; MTX, methotrexate; RA, rheumatoid arthritis; RTX, rituximab; SSZ, sulfasalazine; TCZ, tocilizumab; TOF, tofacitinib. ^a^Infliximab, golimumab, and rituximab are indicated for RA only when administered in combination with MTX. Monitoring frequency should follow that of the recommendations for MTX.
